# Does Timing Matter? Impact of Time to Surgery on Pathologic Response in Gastric Cancer Patients Treated with Perioperative FLOT

**DOI:** 10.3390/medicina62061080

**Published:** 2026-06-02

**Authors:** Bengü Dursun, Seher Kaya, Ferit Aydın, Ata Türker Arıkök

**Affiliations:** 1 Department of Medical Oncology, Ankara Etlik City Hospital, Ankara 06170, Turkey; drkayase@gmail.com; 2 Department of Surgical Oncology, Ankara Etlik City Hospital, Ankara 06170, Turkey; drferita@gmail.com; 3 Department of Pathology, Ankara Etlik City Hospital, Ankara 06170, Turkey; atalina2005@yahoo.com

**Keywords:** gastric cancer, FLOT, neoadjuvant chemotherapy, time to surgery, pathological response, tumor regression grade

## Abstract

*Background and Objectives*: The optimal timing of surgery after preoperative fluorouracil, leucovorin, oxaliplatin and docetaxel (FLOT) chemotherapy (ChT) in gastric cancer (GC) remains unclear. We aimed to evaluate the association between time to surgery (TTS) and pathological as well as survival outcomes in patients treated with perioperative FLOT. *Materials and Methods*: This retrospective cohort study included 76 patients with locally advanced gastric or gastroesophageal junction (GEJ) adenocarcinoma who underwent curative-intent surgery after preoperative FLOT at a single tertiary center. TTS was defined as the interval between completion of preoperative FLOT and surgery. Patients were categorized into two groups according to TTS: ≤4 weeks and >4 weeks. Pathological response (PR) was assessed using the Becker tumor regression grading system. The primary endpoint was PR, including tumor regression grade and pathological complete response (pCR). Secondary endpoint was overall survival (OS). *Results*: The median TTS was 31 days (IQR, 21–47). Forty-five (59.2%) patients underwent surgery within 4 weeks. Favorable PR was more frequently observed in the ≤4-week group. Becker TRG 0–1 was significantly more common among patients undergoing surgery within 4 weeks compared with those undergoing surgery after more than 4 weeks (26.2% vs. 6.5%, *p* = 0.03). Similarly, pCR was observed exclusively in the ≤4-week group (14.3% vs. 0%, *p* = 0.02). ypN0 status was numerically higher in the ≤4-week group (54.7% vs. 32.2%, *p* = 0.05). Postoperative complication rates did not differ significantly between groups (4.8% vs. 12.9%, *p* = 0.17). In multivariable Cox regression analysis, TTS was not independently associated with OS, whereas poor tumor differentiation remained an independent predictor of worse survival (HR 2.57, 95% CI 1.17–5.63, *p* = 0.01). *Conclusions*: Among patients treated with preoperative FLOT, surgery within 4 weeks was associated with improved PR without an apparent increase in postoperative morbidity. However, earlier surgery was not independently associated with improved OS. These findings suggest that prolonged delay after preoperative FLOT may not be necessary in clinically recovered patients and support the need for prospective multicenter studies to define the optimal surgical interval in FLOT-treated GC.

## 1. Introduction

Gastric cancer (GC) remains a major global health burden with many patients presenting with locally advanced but potentially resectable disease [1]. In this setting, multimodal treatment has become the cornerstone of curative-intent management. The FLOT4-AIO trial demonstrated that perioperative fluorouracil, leucovorin, oxaliplatin and docetaxel (FLOT) improved survival outcomes compared with older epirubicin-based regimens, positioning FLOT as a preferred perioperative regimen [2,3]. Beyond its survival benefit, neoadjuvant chemotherapy (NACT) provides an opportunity to assess tumor chemosensitivity before definitive surgery. Pathologic response (PR) after preoperative treatment, commonly evaluated using tumor regression grading systems such as Mandard or Becker classifications, has been increasingly recognized as an important prognostic marker in GC [4,5]. In patients treated with FLOT, improved tumor regression and PR rates have reinforced the clinical relevance of pathological endpoints as surrogate measures of treatment activity [6].

In routine practice, surgery is often scheduled after clinical recovery from chemotherapy (ChT)-related toxicity, optimization of nutritional and performance status and logistical coordination between medical oncology and surgical teams. Many clinical protocols and retrospective studies have commonly used an interval of approximately 4–6 weeks after NACT, but this timing is not supported by definitive prospective evidence [7]. Although a longer interval may allow further tumor regression and improve PR, excessive delay may be associated with disease progression [8]. Also, very early surgery may occur before maximal histological regression has developed or before adequate recovery from systemic therapy has been achieved.

The interval between NACT and surgery represents an important component of perioperative management, but its impact on PR and survival outcomes in FLOT-treated GC remains unclear. Defining the most appropriate surgical interval may help optimize multidisciplinary treatment strategies by balancing recovery from ChT-related toxicity, surgical feasibility and oncologic efficacy. Therefore, in the present study, we investigated the association between time to surgery and pathological as well as survival outcomes in patients with gastric adenocarcinoma treated with perioperative FLOT.

## 2. Methods

### 2.1. Study Design and Patient Population

This retrospective cohort study included 76 patients with histologically confirmed gastric or gastroesophageal junction (GEJ) adenocarcinoma who received preoperative FLOT followed by curative-intent surgical resection between June 2019 and January 2026. Eligible patients were those with non-metastatic, locally advanced disease who received at least one cycle of preoperative FLOT and subsequently underwent gastrectomy. Patients with GEJ tumors were included if they were managed within a GC perioperative FLOT and underwent curative-intent gastrectomy. Esophageal adenocarcinomas requiring esophagectomy were not included. Siewert classification was not systematically available for all GEJ tumors in the retrospective database; therefore, a Siewert-specific analysis of tumor location and resection type could not be performed. Clinical, pathological, treatment-related, surgical and follow-up data were retrospectively obtained from institutional medical records, including archived records from the predecessor institution that was later relocated to Ankara Etlik City Hospital.

Time to surgery (TTS) was defined as the interval between the last day of preoperative FLOT and the date of surgical resection. Patients were categorized into two groups according to this interval: those who underwent surgery within 4 weeks and those who underwent surgery after more than 4 weeks. In routine practice, the timing of surgery was determined through multidisciplinary clinical assessment by the surgical oncology and medical oncology teams, taking into account recovery from preoperative FLOT, treatment-related toxicity, surgical fitness, operating room availability and multidisciplinary tumor board recommendations when applicable. The 4-week cut-off was not prospectively predefined before data collection. It was selected as a pragmatic and clinically interpretable threshold reflecting common surgical scheduling practice after completion of neoadjuvant ChT, as well as the observed distribution of TTS in our cohort. This categorization was used to evaluate whether a delay beyond 4 weeks was associated with pathological response or survival outcome. The number of preoperative cycles, treatment delay, dose reduction and severe treatment-related toxicity were recorded. Postoperative systemic treatment, including completion of perioperative FLOT or use of alternative adjuvant regimens, was administered at the discretion of the treating physicians according to postoperative recovery, pathological findings, toxicity profile, and multidisciplinary tumor board recommendations. All patients underwent D2 lymphadenectomy, thereby minimizing heterogeneity in surgical management and allowing more consistent evaluation of pathological and oncologic outcomes. Resection margin status was classified as R0, R1 or R2 according to standard pathological criteria. Severe postoperative complications (anastomotic leakage, postoperative bleeding, bowel obstruction and surgical site infection) were recorded from surgical and postoperative follow-up documentation.

Resected specimens were assessed by an institutional pathologist using standard histopathological criteria. Pathological assessment was performed as part of routine clinical practice. The pathologists were not formally blinded to the timing group, and no centralized second pathology review was performed. Pathological variables were extracted from the original pathology reports. For variables with missing data, patients were excluded only from the corresponding variable-specific analysis, and no imputation was performed. Percentages were calculated according to the number of patients with available data for each variable. Pathological variables included ypT stage, ypN status, resection margin status, pathological complete response (pCR) and tumor regression grade. Tumor regression was evaluated using the Becker tumor regression grading system. For analytic purposes, Becker TRG 0–1 was categorized as good pathological response, whereas Becker TRG 2–3 was categorized as poor pathological response. pCR was defined as the absence of residual viable tumor in the resected primary tumor specimen. Clinical-to-pathological downstaging was assessed by comparing pretreatment clinical staging with postoperative pathological staging.

The primary endpoint was the association between TTS and PR after preoperative FLOT. Pathological outcomes included Becker tumor regression grade, pCR, ypT stage, ypN status, resection margin status and postoperative complications. The secondary endpoint was OS, defined as the interval from initiation of preoperative FLOT to death from any cause. Patients who remained alive at the last follow-up were censored at the date of last known contact.

This study was approved by the Institutional Review Board of the Clinical Research Ethics Committee of Etlik City Hospital [approval number: AEŞH-BADEK1-2026-287; Date of approval: 8 April 2026]. The requirement for informed consent was waived by the Etlik City Hospital Ethics Committee due to the retrospective design of the study. All methods were performed in accordance with the relevant guidelines and regulations, including the Declaration of Helsinki.

### 2.2. Statistical Analysis

Continuous variables were summarized as median and interquartile range and categorical variables were summarized as number and percentage. Comparisons between the ≤4-week and >4-week groups were performed using the Mann–Whitney U test for continuous variables and the chi-square test or Fisher’s exact test for categorical variables, as appropriate. Percentages were calculated according to the number of patients with available data for each variable. Overall survival (OS) was estimated using the Kaplan–Meier method, and survival distributions were compared using the log-rank test. Univariable Cox proportional hazards regression analysis was performed to identify factors associated with OS. Variables with a *p*-value < 0.10 in univariable analysis were considered candidates for the multivariable Cox proportional hazards model. This threshold was selected to avoid excluding potentially relevant prognostic variables at the initial screening stage, particularly given the exploratory nature of the study and the limited number of survival events. Time to surgery was included in the multivariable Cox model regardless of its univariable *p*-value because it represented the main exposure of interest and the primary clinical question of the study. Becker tumor regression grade was not included in the multivariable model despite its univariable association with OS because no death events occurred among patients with good tumor regression, resulting in complete separation and unstable parameter estimation. The final model included comorbidity, tumor differentiation, TTS and ypN status. Primary tumor location was not retained in the final model to preserve model parsimony; however, a sensitivity model including primary tumor location was performed and yielded similar results for the association between TTS and OS. Hazard ratios (HR) with 95% confidence intervals were reported. All statistical tests were two-sided, and *p*-values < 0.05 were considered statistically significant. Statistical analyses were performed using IBM SPSS Statistics for Windows, version 25.0.

## 3. Results

### Baseline Characteristics

A total of 76 patients with histologically confirmed gastric or GEJ adenocarcinoma who underwent curative-intent surgery after preoperative FLOT were included. The median age was 61 years (IQR, 54–69) and the median BMI was 23.3 kg/m^2^ (IQR, 19.7–27.5). The cohort was predominantly male (n = 67, 88.2%) and most patients had gastric primary tumors (n = 57, 75.0%) rather than GEJ tumors (n = 19, 25.0%). Clinically node-positive disease was present in 70 patients (92.1%), and clinical T4 disease was observed in 47 patients (61.8%). Most patients received four cycles of preoperative FLOT (n = 50, 65.7%), whereas a smaller proportion received either fewer or more cycles. Dose reduction or treatment delay occurred in 20 patients (26.3%) and grade 3–4 toxicity was observed in 9 patients (11.8%). Completion of the planned perioperative FLOT regimen was achieved in 51 patients (67.1%). The baseline clinicopathological and treatment-related characteristics of the overall cohort are presented in Table 1.

The median TTS interval after completion of neoadjuvant FLOT was 31 days (IQR, 21–47). Using 4 weeks as the cut-off, the median TTS was 23 days (IQR, 19–28) in the ≤4-week group and 49 days (IQR, 38–63) in the >4-week group. Baseline demographic, clinicopathological and treatment-related characteristics were largely comparable between the two groups, with no statistically significant differences in age, BMI, comorbidity, ECOG PS, primary tumor location, tumor differentiation, cT stage, clinical nodal status, number of preoperative FLOT cycles or dose reduction/treatment delay (all *p* > 0.05). Grade 3–4 toxicity was numerically more frequent among patients undergoing surgery after more than 4 weeks, although this difference did not reach statistical significance (19.4% vs. 6.7%, *p* = 0.09) (Table 2).

Pathological outcomes favored earlier surgery after preoperative FLOT. Patients in the ≤4-week group had a higher proportion of ypT0–T2 disease than those in the >4-week group (47.6% vs. 32.2%), although this difference was not statistically significant. ypN0 status was also more frequent in the ≤4-week group (54.7% vs. 32.2%), with borderline statistical significance. R0 resection rates were numerically higher in the ≤4-week group but did not differ significantly between groups. A significant association was observed between TTS and tumor regression. Becker TRG 0–1 was more frequent among patients undergoing surgery within 4 weeks compared with those undergoing surgery after more than 4 weeks (26.2% vs. 6.5%), whereas Becker TRG 2–3 predominated in the delayed-surgery group. pCR was observed exclusively in the ≤4-week group (14.3% vs. 0%). The incidence of severe postoperative complications did not differ significantly between the two groups (*p* = 0.17). These results suggest that surgery within 4 weeks after preoperative FLOT was associated with improved PR, without an apparent increase in postoperative morbidity (Table 3).

**Figure 1:**
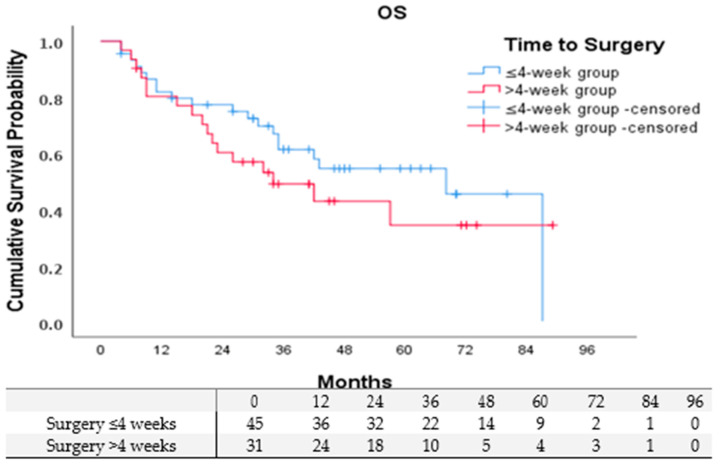
Overall Survival According to Time to Surgery After Neoadjuvant FLOT.

The median follow-up time was 35 months (IQR, 16–52) in the ≤4-week group and 30 months (IQR, 15–42) in the >4-week group, with no statistically significant difference between groups (*p* = 0.30). Kaplan–Meier analysis did not demonstrate a statistically significant difference in OS between patients undergoing surgery within 4 weeks and those undergoing surgery after more than 4 weeks. Although median OS was numerically longer in the ≤4-week group than in the >4-week group (68.0 vs. 34.0 months), this difference was not statistically significant (log-rank *p* = 0.317) (Figure 1). In univariable Cox regression analysis, poor tumor differentiation and poor tumor regression according to Becker classification were associated with inferior OS. Comorbidity and ypN positivity showed borderline associations with survival, whereas time to surgery beyond 4 weeks was not significantly associated with OS. In the multivariable Cox model, poor tumor differentiation remained independently associated with worse OS (*p* = 0.01). In contrast, time to surgery beyond 4 weeks was not independently associated with OS after adjustment for clinically relevant covariates (Table 4). Although Becker TRG was significantly associated with OS in univariable analysis, it was not included in the multivariable model because no death events occurred among patients with good tumor regression, resulting in complete separation and unstable parameter estimation. TTS was retained in the model because it was the main exposure of interest.

## 4. Discussion

The optimal timing of surgery after preoperative FLOT remains an unresolved issue in contemporary GC management. In this retrospective cohort study, we evaluated this question in a homogeneous population of patients with gastric or GEJ adenocarcinoma treated exclusively with perioperative FLOT. To our knowledge, this is among the first studies specifically designed to assess the association between TTS and pathological and survival outcomes in an entirely FLOT-treated cohort. Our findings suggest that surgery within 4 weeks after FLOT had higher rates of favorable tumor regression and pCR compared with surgery after more than 4 weeks. Importantly, approximately 70% of patients in the ≤4-week group had received at least four cycles of preoperative FLOT, indicating that earlier surgery did not simply reflect abbreviated preoperative treatment exposure. However, this apparent pathological advantage did not translate into a statistically significant OS benefit. In multivariable analysis, poor tumor differentiation, rather than TTS, remained independently associated with worse survival.

Our findings are consistent with the hypothesis that a prolonged interval after cytotoxic ChT does not necessarily improve pathological regression in GC [7]. In contrast to neoadjuvant chemoradiotherapy, where delayed surgery may allow for ongoing radiation-induced tumor regression, cytotoxic ChT may exert its maximal effect closer to the completion of treatment [9]. Prolonging the interval may allow for the recovery of normal tissues, but it may also permit tumor cell repopulation in non-completely responding tumors [10,11]. This biological distinction may explain why data from rectal or esophageal chemoradiotherapy cannot be directly extrapolated to GC treated with ChT alone. In our cohort, surgery within 4 weeks was associated with higher Becker TRG 0–1 and pCR rates, suggesting that earlier resection after FLOT may capture a more favorable window of treatment-induced tumor regression before potential biological recovery of residual disease.

Previous Asian cohorts have reported conflicting results regarding delayed surgery after NACT. Liu et al. found that an interval longer than 6 weeks was associated with higher odds of PCR, although this did not translate into improved OS or DFS [12]. Conversely, Wang et al. suggested that a 3–5-week interval may represent a more favorable window for survival, with no clear pathological or morbidity advantage from longer delays [13]. However, both studies predominantly included SOX/XELOX or CapeOX-based regimens rather than FLOT, limiting their direct applicability to contemporary FLOT-treated populations. Consistent with our findings, a perioperative cohort in which approximately 21% of patients received FLOT showed that earlier surgery was associated with higher major pathological response rates, without an increase in postoperative morbidity [14]. Because FLOT has distinct efficacy and toxicity profiles, surgical timing data derived from older or non-FLOT regimens may not fully apply to current practice. In this regard, our study addresses a clinically relevant gap by evaluating a modern, relatively homogeneous FLOT-treated cohort. Furthermore, all patients underwent D2 lymphadenectomy, strengthening the internal consistency of surgical management and reducing the likelihood that differences in pathological or survival outcomes were driven by variability in lymphadenectomy extent. Collectively, these findings argue against routinely delaying surgery after completion of preoperative FLOT in the expectation of achieving superior pathological regression.

Despite the higher PR rate observed in the ≤4-week group, TTS was not significantly associated with OS. This may be explained by the multifactorial nature of survival after perioperative treatment in GC. Previous studies have shown that histopathological tumor regression is prognostically relevant, but its survival impact may be attenuated after adjustment for stronger determinants such as ypN status, margin status and pathological stage [15,16]. In particular, post-treatment nodal status has repeatedly been reported as one of the most robust predictors of survival after NACT, sometimes outweighing the prognostic effect of tumor regression itself [17,18]. Similarly, R0 resection and the ability to complete perioperative or postoperative systemic therapy remain major determinants of long-term outcome [2,19]. In our cohort, poor tumor differentiation was the only independent predictor of worse OS, supporting the dominant role of intrinsic tumor biology. GC is characterized by marked histologic and molecular heterogeneity and treatment response may be influenced by epithelial–mesenchymal transition-related phenotypes, altered cell–cell adhesion and tumor plasticity [20]. The independent association between poor differentiation and OS in our cohort further supports the concept that histologic differentiation reflects underlying biological diversity rather than morphology alone [21]. Although Becker TRG was associated with OS in univariable analysis, it could not be included in the multivariable model because no death events occurred among patients with good tumor regression, resulting in complete separation. This finding is biologically consistent with the favorable prognosis of marked tumor regression, but also reflects the statistical limitations of a small cohort with limited survival events.

The lack of a statistically significant survival difference should therefore not be interpreted as evidence that surgical timing is irrelevant. Rather, our findings suggest that earlier surgery may be associated with improved PR, whereas the survival impact of this association remains uncertain. Importantly, the absence of an independent association between TTS and OS indicates that surgery within 4 weeks should not be interpreted as necessarily superior in terms of long-term survival. The observed improvement in Becker TRG and pCR should therefore be regarded as an exploratory pathological finding rather than as evidence of a definitive survival advantage. This distinction is clinically important because PR is an early and biologically meaningful endpoint, but it is not a perfect surrogate for OS in GC. Survival after perioperative treatment for GC is multifactorial and may be influenced by intrinsic tumor biology, ypN status, margin status, postoperative recovery, completion of perioperative chemotherapy, comorbidities, and subsequent treatment [18]. In addition, the relatively small sample size and limited number of survival events in the present cohort may have reduced the statistical power to detect modest survival differences. Importantly, postoperative treatment completion remains challenging in real-world FLOT-treated populations [22,23]. In our cohort, only 67.1% of patients completed the planned perioperative FLOT regimen, consistent with the broader clinical experience that postoperative ChT delivery is frequently limited by surgical recovery, toxicity and patient fitness. Thus, the timing of surgery should be considered as one component of a broader perioperative strategy rather than as an isolated determinant of outcome.

Several limitations should be acknowledged. The retrospective, single-center design introduces the possibility of selection bias and residual confounding. Although baseline characteristics were broadly comparable between the timing groups, the timing of surgery in routine practice may have been influenced by unmeasured patient-, treatment-, and system-related factors, including chemotherapy-related toxicity, nutritional status, frailty, clinical recovery, comorbidities, radiologic response, hospital logistics, multidisciplinary scheduling, and surgeon preference. Therefore, the association between surgery within 4 weeks and improved pathological response should be interpreted as exploratory and hypothesis-generating rather than as evidence of a definitive causal threshold. In addition, the 4-week cut-off was clinically pragmatic and was not prospectively validated; accordingly, it should not be regarded as a definitive or universally applicable threshold. Moreover, the relatively small sample size limited statistical power, particularly for survival analyses and multivariable modeling. The relatively low number of pCR events should also be considered when interpreting subgroup comparisons. Becker TRG and pCR, although clinically meaningful pathological endpoints, may not fully capture the invasive biological behavior of residual GC. Contemporary pathological studies increasingly emphasize the relevance of tumor budding, stromal remodeling and tumor–stroma interface patterns, which may influence both regression assessment and prognosis [24,25]. These features were not systematically evaluated in the present retrospective cohort and should be considered in future prospective studies. Finally, pathological assessment was performed in routine clinical practice rather than through centralized or blinded second pathology review, although all specimens were evaluated according to standard histopathological criteria.

## 5. Conclusions

In patients with locally advanced gastric or GEJ adenocarcinoma treated with preoperative FLOT, surgery performed within 4 weeks was associated with higher rates of favorable tumor regression and pCR without an apparent increase in postoperative morbidity. However, this pathological advantage did not translate into an independent OS benefit; therefore, these findings should be interpreted cautiously in view of the retrospective design, limited sample size and potential selection bias. Rather than establishing a definitive optimal threshold, our findings suggest that prolonged delay after completion of preoperative FLOT may not be required in clinically recovered patients. Because TTS was not independently associated with OS, the observed pathological advantage of earlier surgery should be interpreted as exploratory and should not be considered evidence of a survival benefit. Given the retrospective nature of the present study and the limited availability of FLOT-specific timing data, prospective multicenter studies focused exclusively on modern perioperative FLOT-treated cohorts are warranted to define the optimal surgical interval and to clarify whether improved PR can ultimately translate into durable survival benefit.

## Figures and Tables

**Table 1:** Baseline Clinicopathological Characteristics of Patients.

Variable	n, (%)	Variable	n, (%)
Age, years, median (IQR)	61 (54–69)	Clinical nodal status	
BMI, median (IQR), (kg/m^2^)	23.3 (19.7–27.5)	Positive	70 (92.1)
Sex		Negative	6 (7.9)
Female	9 (11.8)	Number of preoperative FLOT cycles	
Male	67 (88.2)	1–3	8 (10.5)
Presence of Comorbidity		4	50 (65.7)
Yes	36 (47.3)	5–8	18 (23.6)
No	40 (52.7)	Dose reduction or treatment delay	
ECOG PS		Yes	20 (26.3)
0	18 (23.7)	No	56 (73.7)
1	54 (71.1)	Grade 3–4 toxicity	
2	4 (5.3)	Yes	9 (11.8)
Primary tumor location		No	67 (88.2)
GEJ	19 (25)	Type of adjuvant chemotherapy	
Gastric	57 (75)	None	5 (6.6)
Tumor differentiation		FLOT	51 (67.1)
Well	13 (17.1)	FOLFOX/CAPOX	10 (13.2)
Moderate	27 (35.5)	Fluoropyrimidine monotherapy	1 (1.3)
Poor	15 (19.8)	Unknown	9 (11.8)
Unknown	21 (27.6)	Adjuvant radiotherapy	
Clinical T stage		Yes	2 (2.6)
cT2	3 (3.9)	No	73 (96.1)
cT3	26 (34.2)	Unknown	1 (1.3)
cT4	47 (61.8)		

Abbreviations: BMI, body mass index; IQR, interquartile range; ECOG PS, Eastern Cooperative Oncology Group performance status; GEJ, gastroesophageal junction; FLOT, fluorouracil, leucovorin, oxaliplatin, and docetaxel; FOLFOX, fluorouracil, leucovorin, and oxaliplatin; CAPOX, capecitabine and oxaliplatin.

**Table 2:** Comparison of baseline and treatment characteristics according to time to surgery.

Variable	Surgery ≤4 Weeks n: 45	Surgery >4 Weeks n: 31	*p* Value
Age, years, median (IQR)	61 (53–68)	65 (52–72)	0.38
BMI, median (IQR), (kg/m^2^)	23.5 (19.9–27.7)	23 (20.3–26.4)	0.96
Presence of Comorbidity			0.29
Yes	23 (51.1)	12 (38.7)	
No	21 (46.6)	18 (58.0)	
ECOG PS			0.85
0	11 (24.4)	7 (22.6)	
1–2	34 (75.6)	24 (77.4)	
Primary tumor location			0.34
GEJ	13 (28.9)	6 (19.4)	
Gastric	32 (71.1)	25 (80.6)	
Tumor differentiation			
Well/Moderate	20 (44.4)	20 (64.5)	0.82
Poor	7 (15.5)	8 (25.8)	
Clinical T stage			0.57
cT2–cT3	16 (35.6)	13 (41.9)	
cT4	29 (64.4)	18 (58.1)	
Clinical nodal status			0.65
Positive	42 (93.3)	28 (90.3)	
Negative	3 (6.7)	3 (9.7)	
Number of preoperative FLOT cycles			0.71
1–4	35 (77.8)	23 (74.2)	
5–8	10 (22.2)	8 (25.8)	
Dose reduction or treatment delay			0.69
Yes	15 (33.3)	9 (29)	
No	30 (66.7)	22 (71)	
Grade 3–4 toxicity			0.09
Yes	3 (6.7)	6 (19.4)	
No	42 (93.3)	25 (80.6)	
Completion of planned perioperative FLOT			0.37
Yes	32 (71.1)	19 (61.3)	
No	13 (28.9)	12 (38.7)	

Abbreviations: BMI, body mass index; ECOG PS, Eastern Cooperative Oncology Group performance status; FLOT, fluorouracil, leucovorin, oxaliplatin, and docetaxel; GEJ, gastroesophageal junction; IQR, interquartile range. Note: Data are shown as median (IQR) or n (%). Percentages were calculated according to the available data for each variable. Missing data were excluded from percentage calculations.

**Table 3:** Pathological and surgical outcomes according to time to surgery.

Variable	Surgery ≤4 Weeks n: 42	Surgery >4 Weeks n: 31	*p* Value
ypT stage			0.18
ypT0–T2	20 (47.6)	10 (32.2)	
ypT3–T4	22 (52.4)	21 (67.8)	
ypN status			0.05
ypN0	23 (54.7)	10 (32.2)	
ypN+	19 (45.3)	21 (67.8)	
Resection margin			0.39
R0	38 (90.4)	26 (83.8)	
R1/2	4 (9.5)	5 (16.1)	
Tumor regression grade, Becker			0.03
TRG 0–1	11 (26.2)	2 (6.5)	
TRG 2–3	31 (73.8)	29 (93.5)	
Pathological complete response			0.02
Yes	6 (14.3)	0 (0)	
No	36 (85.7)	31 (100)	
Severe postoperative complications			0.17
Yes	2 (4.76)	4 (12.9)	
No	40 (95.2)	27 (87.1)	

Abbreviations: pCR, pathological complete response; R0, microscopically margin-negative resection; R1, microscopically margin-positive resection; R2, macroscopically margin-positive resection; TRG, tumor regression grade; ypN, pathological nodal stage after neoadjuvant treatment; ypT, pathological tumor stage after neoadjuvant treatment. Note: Data are presented as n (%). Percentages were calculated according to the available data for each variable. Becker TRG 0–1 was considered a good pathological response, whereas TRG 2–3 was considered a poor pathological response.

**Table 4:** Univariable and Multivariable Cox Regression Analyses for Overall Survival.

Variable	Univariate HR (95% Cl)	*p*-Value	Multivariate HR (95% CI)	*p*-Value
Age	1.01 (0.97–1.04)	0.48		
BMI	1.01 (0.92–1.08)	0.99		
ECOG PS (1 vs. 0)	1.27 (0.61–2.65)	0.50		
Comorbidity (yes vs. no)	1.90 (0.96–3.75)	0.06	1.90 (0.89–4.08)	0.09
Primary tumor location (GEJ vs. gastric)	0.47 (0.19–1.13)	0.10		
Tumor differentiation (poor vs. well-moderate)	2.71 (1.30–5.63)	0.008	2.57 (1.17–5.63)	0.01
Number of preoperative FLOT cycles (≤4; >4)	1.11 (0.52–2.36)	0.78		
Time to surgery (>4 week vs. ≤4week)	1.38 (0.72–2.65)	0.32	1.82 (0.85–3.87)	0.12
ypT stage (T3–4; T1–2)	1.60 (0.76–3.36)	0.21		
ypN stage (positive; negative)	2.00 (0.96–4.14)	0.06	1.29 (0.57–2.91)	0.53
Clinical-to-pathological downstaging (yes vs. no)	0.70 (0.35–1.42)	0.33		
Tumor regression grade, Becker (TRG 2–3 vs. TRG 0–1)	4.56 (1.08–19.12)	0.03		
Completion of perioperative FLOT (no vs. yes)	1.61 (0.80–3.23)	0.18		

Abbreviations: BMI, body mass index; CI, confidence interval; ECOG PS, Eastern Cooperative Oncology Group performance status; FLOT, fluorouracil, leucovorin, oxaliplatin, and docetaxel; GEJ, gastroesophageal junction; HR, hazard ratio; ypT, post-neoadjuvant pathological tumor stage; ypN, post-neoadjuvant pathological nodal stage; TRG, tumor regression grade. Footnotes: Hazard ratios were estimated using Cox proportional hazards regression analysis. Variables with a *p*-value < 0.10 in univariable analysis, together with time to surgery as the main variable of interest, were considered for inclusion in the multivariable model. The final multivariable model included comorbidity, tumor differentiation, time to surgery, and ypN stage. Becker tumor regression grade was not included in the multivariable model because no death events occurred among patients with good tumor regression, resulting in complete separation and unstable parameter estimation.

## Data Availability

The datasets generated and/or analyzed during the current study are available from the corresponding author on reasonable request.
